# Temporal Patterns and Treatment Associations in Complications Following Hip Arthroplasty

**DOI:** 10.3390/diagnostics15070815

**Published:** 2025-03-23

**Authors:** Rolland Fazakas, Laura Ioana Bondar, Csongor Toth, Caius Calin Miuța, Iosif Ilia, Corina Dalia Toderescu, Alexandru Pop

**Affiliations:** 1 Doctoral School of Medicine, “Vasile Goldiș” Western University of Arad, 310048 Arad, Romania; fazakas.roland@uvvg.ro (R.F.); pop.alexandru@uvvg.ro (A.P.); 2 Department of Biology and Life Sciences, Faculty of Medicine, “Vasile Goldiș” Western University of Arad, 310048 Arad, Romania; 3 Doctoral School of Biomedical Sciences, University of Oradea, 410087 Oradea, Romania; csongor.toth@uav.ro; 4 Faculty of Physical Education and Sport, “Aurel Vlaicu” University of Arad, 310130 Arad, Romania; iosif.ilia@uav.ro; 5 Faculty of Medicine, “Victor Babeș” University of Medicine and Pharmacy, 300041 Timișoara, Romania; corina.toderescu@umft.ro; 6 Department of General Medicine, Faculty of Medicine, “Vasile Goldiș” Western University of Arad, 310048 Arad, Romania

**Keywords:** antibiotics, complications, dislocation, fracture, hip arthroplasty, implant loosening, infection, mechanical failures, treatment

## Abstract

**Background and Objectives:** Hip arthroplasty is commonly performed to enhance mobility and quality of life in patients with severe joint degeneration. However, post-surgery complications such as infections, dislocations, and mechanical failures remain prevalent and vary over time. This study examines the relationship between time intervals post-surgery and the occurrence of complications and explores the associations between specific treatment modalities and complications. It also investigates temporal patterns of infectious and mechanical complications to inform more effective post-surgery care. **Materials and Methods:** A retrospective cohort study was conducted on hip arthroplasty patients to analyze the occurrence and distribution of complications across medium-term (1–5 years) and long-term (≥6 years) intervals. Treatment modalities, including joint debridement, lavage, antibiotics, and mechanical interventions, were analyzed for their association with complications. Chi-Square tests were used, with significance set at *p* < 0.05. **Results:** A significant association was found between time intervals and complications (χ^2^ = 58.149, df = 19, *p* < 0.001). Infections were more prevalent in the medium-term, while mechanical complications such as dislocation, implant loosening, and periprosthetic fractures were more common in the long-term. Antibiotics were strongly linked to infectious complications (χ^2^ = 279.000, *p* < 0.001), and mechanical treatments were associated with fractures and dislocations. **Conclusions:** The study confirms that the timing of complications post-surgery plays a critical role in their occurrence. Specific complications become more prevalent at different intervals, emphasizing the need for tailored treatment strategies. Antibiotics for infections and mechanical interventions for fractures and dislocations should be adjusted based on timing. These findings highlight the importance of time-specific post-surgery care and suggest areas for further research on long-term strategies and risk factors.

## 1. Introduction

Hip arthroplasty, a widely performed surgical procedure for treating severe arthritis and joint degeneration, is recognized as an effective means of restoring mobility and improving the quality of life for patients [1,2]. The procedure involves replacing the hip joint with a prosthetic implant to alleviate pain, enhance function, and enable individuals to resume normal daily activities [3,4]. Advancements in surgical techniques, prosthetic designs, and perioperative care have significantly improved outcomes [5,6]. However, despite these improvements, complications following hip arthroplasty remain a significant challenge, affecting patient quality of life and placing a considerable burden on healthcare systems [7,8].

Complications such as infections, dislocations, mechanical failures, and periprosthetic fractures, can occur at various stages of recovery, with distinct temporal patterns [9,10]. Infections, for instance, are more common in the early postoperative period, while mechanical failures including dislocation, implant loosening, and periprosthetic fractures typically arise in the long term [11,12]. Identifying these temporal patterns is crucial for optimizing patient care, enabling clinicians to predict and intervene proactively to prevent adverse outcomes [13,14].

Despite extensive research on hip arthroplasty complications, significant gaps remain in understanding how these complications evolve over time and how treatment modalities influence their occurrence [15,16]. Limited studies have analyzed the temporal distribution of complications across medium-term (1–5 years) and long-term (≥6 years) postoperative intervals, and the effectiveness of treatments such as antibiotics, joint debridement, and mechanical interventions in addressing specific complications remains underexplored [17,18,19]. These gaps hinder the development of time-sensitive, evidence-based care strategies tailored to the evolving needs of hip arthroplasty patients.

To address these gaps, this study examines the temporal evolution of hip arthroplasty complications and their associations with specific treatment modalities. While early antibiotic interventions may reduce the infection risks, long-term implant survival often hinges on addressing mechanical complications such as dislocations and fractures [20,21]. Additionally, the timing and effectiveness of interventions such as joint debridement and mechanical treatments, remain topics of ongoing debate [22,23]. By analyzing patterns of infections, dislocations, fractures, and other complications across medium- and long-term intervals, this research provides actionable insights for improving postoperative management.

This study aims to analyze the temporal distribution of hip arthroplasty complications, evaluate the associations between treatment modalities and specific complication types, and provide evidence-based recommendations for postoperative care. By identifying time-sensitive risks and effective interventions, these findings can inform clinical decision-making, improve long-term patient outcomes, and reduce complication-related morbidity.

## 2. Materials and Methods

### 2.1. Study Design and Setting

This retrospective study was conducted at the Arad Clinical Emergency County Hospital, Romania, a prominent prosthetic center since 1970. The research examined complications associated with primary total hip prostheses within the Orthopedics-Traumatology department. Data was collected from hospital archives for patients hospitalized between 1 January 2000, and 31 December 2019, and analyzed retrospectively. The study aims to assess the frequency and distribution of complications related to total hip arthroplasties over medium (1–5 years) and long-term (≥6 years) intervals following implantation.

Although standardized definitions in the literature categorize postoperative intervals differently (short-term: <2 years; medium-term: 2–10 years; and long-term: ≥10 years) [24]. This study specifically defined the medium-term interval as 1–5 years and the long-term as ≥6 years post-surgery. This decision was intentionally made to distinctly capture complications typically seen after the initial early recovery period, which is well-documented, versus those complications emerging or significantly evolving in subsequent periods. Thus, this study provides targeted insights into medium- and long-term complications, addressing a gap in existing literature concerning these specific intervals.

### 2.2. Study Population

The study population consisted of 279 patients hospitalized in the Orthopedics-Traumatology department during the specified period, who underwent primary total hip arthroplasty. This section outlines the criteria used to determine eligibility for inclusion or exclusion from the study.

#### 2.2.1. Inclusion Criteria

Patients were eligible for inclusion if they were adults (≥18 years) who underwent primary total hip arthroplasty. Only patients with documented post-surgical complications occurring within either the medium-term (1–5 years) or long-term (≥6 years) following surgery were considered. Additionally, complete medical records with sufficient clinical details to identify and classify complications were required for inclusion.

#### 2.2.2. Exclusion Criteria

Patients were excluded if they had no documented complications following surgery or if their complications were unrelated to hip arthroplasty. Cases with incomplete medical records or missing complication data were also excluded from the study.

### 2.3. Data Collection Instruments

Data was extracted from hospital medical records, which provided detailed information on the patient’s age, comorbidities, years since the primary hip prosthesis implantation, treatments received, and documented complications. The analysis focused on various complications, including chronic fistulized arthritis, implant loosening, dislocations, infections, fractures, heterotopic ossifications, periprosthetic fractures, hematomas, recurrent dislocations, metallosis, and Non-Articular Collapsing Fractures (NACF).

Data on comorbidities were also collected and analyzed. These included diabetes mellitus and other metabolic conditions such as hypothyroidism, hyperthyroidism, dyslipidemia, and metabolic syndrome. These conditions were chosen for their potential impact on post-surgical outcomes, as metabolic imbalances can affect wound healing, infection risk, and overall recovery following total hip arthroplasty. Identifying and classifying these conditions allowed for a more nuanced analysis of their association with documented complications.

Chronic fistulized arthritis was defined as a persistent septic complication characterized by chronic joint infection leading to the formation of a fistula, a pathological connection between the joint space and the skin or another external surface. Unlike general classifications of septic complications into acute (6–12 weeks) and chronic infections, this term specifically refers to cases where a chronic infection progresses to fistula formation, necessitating distinct clinical management. This condition represents a distinct subset of chronic infections, requiring specific clinical attention due to its prolonged course and complex management [25,26,27].

Treatments recorded in this study were categorized into antibiotic use and mechanical interventions, based on their relevance in managing complications.

Antibiotic treatments were documented as part of infection management, with records indicating whether antibiotics were administered. However, detailed information on specific agents, duration, route of administration, or indications was unavailable. The analysis focused solely on the presence or absence of antibiotic use in treating infectious complications.

Mechanical treatments addressed structural complications such as dislocations, fractures, and prosthetic failures. These interventions included dislocation reduction, typically performed under anesthesia and joint stabilization procedures for fractures or instability. Cementation repairs were used for implant loosening, while osteosynthesis with plates, screws, or rods facilitated fracture management. Additionally, prosthetic component revisions, involving partial or complete replacement of failed components, were documented.

Treatment categorization was based on operative reports and discharge summaries, allowing for the analysis of associations between specific interventions and the complications they addressed.

Although this study primarily focuses on primary total hip arthroplasty, revision arthroplasties were included in the analysis to provide a comparative perspective on complication rates. These cases were analyzed to better understand differences in complication distribution across prosthesis types, offering insights into potential risk factors and long-term outcomes associated with hip prostheses. Their inclusion enhances the study’s ability to assess how complications evolve across different surgical interventions.

### 2.4. Ethical Considerations

The study was approved by the Ethics Committee of the Arad Clinical Emergency County Hospital, Romania, under protocol code 81/06.06.2024. Patient confidentiality was ensured by anonymizing all data, with no personal identifying information used in the analysis.

### 2.5. Data Analysis

Data were analyzed using JASP 0.19.2 (University of Amsterdam, The Netherlands). Descriptive statistics were used to summarize patient characteristics, including age, comorbidities, time since surgery, and the occurrence of complications, using frequencies and percentages where appropriate.

Chi-square tests were conducted to examine associations between complication types, including dislocations, infections, fractures, and metallosis, and time intervals since surgery (medium-term: 1–5 years; long-term: ≥6 years). A significance level of *p* < 0.05 was applied to determine statistical significance.

Subgroup analyses compared the distribution of complications across medium- and long-term intervals, providing a more detailed understanding of how complications evolve over time. To assess the strength of associations, Cramér’s V test was applied, with values interpreted as follows: 0 indicating no association, 0.1–0.3 representing a weak association, 0.3–0.5 indicating a moderate association, and values greater than 0.5 reflecting a strong association. This structured statistical approach ensures a reliable and robust interpretation of findings.

### 2.6. Hypotheses of the Study

This study aims to investigate various factors influencing complications in prosthetic joint patients. Based on existing literature and previous research, the following hypotheses have been formulated:

1. Time Intervals Post-Surgery and the Occurrence and Distribution of Complications: It is hypothesized that there will be a significant association between time intervals post-surgery and the occurrence and distribution of complications in prosthetic joint patients. Specifically, complications such as infections are expected to be more prevalent in the medium-term (1–5 years) after surgery, while mechanical complications like dislocation, implant loosening and periprosthetic fractures are anticipated to occur more frequently in the long-term (≥6 years).
2. Association Between Treatment Modalities and Specific Types of Complications: The study hypothesizes that certain treatment modalities, such as joint debridement, lavage, and antibiotics, will show a significant association with specific types of complications, including idiopathic, infectious, mechanical, and traumatic complications. Specifically, antibiotics are expected to be most strongly linked to infectious complications, while mechanical treatments such as reduction and skeletal traction are hypothesized to be associated with mechanical and traumatic complications.
3. Higher Incidence of Infectious Complications in the Medium-Term: It is hypothesized that the incidence of infectious complications will be higher in the medium-term (1–5 years) following prosthetic joint surgery compared to the long-term (≥6 years).
4. Predominant Use of Antibiotics for Infectious Complications: The study hypothesizes that the use of antibiotics will be predominantly associated with infectious complications in prosthetic joint patients.
5. Increase in Mechanical Complications Over Time: It is hypothesized that mechanical complications, such as implant loosening and periprosthetic fractures, will increase as time post-surgery extends. As prosthetic joints undergo wear and tear over time, the likelihood of mechanical failures such as implant loosening and fractures is expected to rise in the long-term (≥6 years).

## 3. Results

### 3.1. Demographics and Descriptive Statistics

The study included 279 patients, with a higher proportion of females (56.27%) than males (43.73%). Most participants (51.26%) were aged 71–92 years, followed by 44.44% in the 51–70 age group, and only 4.3% in the 35–50 age range (Table 1).

### 3.2. Association Between Gender and Complications

The distribution of complications varied by gender (Table 2). Dislocations were the most common complication, occurring more frequently in females (46 cases vs. 21 in males) for Cemented Total Prostheses (CTP) and evenly in Uncemented Total Prostheses (UTP) (11 vs. 12 cases).

Implant loosening (31 cases) and infections (40 cases) were prevalent, with infections evenly distributed between genders. Femur periprosthetic fractures were more frequent in males (21 cases vs. 11 in females), while prosthesis-related fractures were slightly higher in females (8 cases vs. 4 in males).

Recurrent dislocations were more frequent in females (15 cases vs. 5 in males). Less common complications, including periprosthetic metallosis (6 cases), chronic fistulized arthritis (9 cases), and prosthetic NACF (1 case), showed no notable gender differences. Heterotopic ossifications were more common in males (7 of 10 cases).

A chi-square test confirmed a significant association between gender and complications (Χ^2^ = 40.319, df = 19, *p* = 0.003) with a moderate correlation (Cramer’s V = 0.380), indicating that complication patterns differ by gender (Table 3).

### 3.3. Analysis of Complication Distribution and Association with Age Group and Prosthesis Type

The distribution of complications varied by age and prosthesis type (Table 4). CTP had the highest complication rates, with dislocations (67 cases) and infections (40 cases) being the most common. Dislocations were most frequent in the 71–92 age group (43 cases), followed by the 51–70 group (23 cases). Infections followed a similar pattern, with 23 cases in the 71–92 group and 16 in the 51–70 group.

Femur periprosthetic fractures were most frequent in the 51–70 age group (22 cases), totaling 32 cases. Less common complications included chronic fistulized arthritis (5 cases), periprosthetic fractures (12 cases), and recurrent dislocations (20 cases).

Among UTP, dislocation (23 cases) was the most common complication, affecting primarily the 51–70 age group (15 cases). Heterotopic ossifications (7 cases) occurred exclusively in the 71–92 group.

Total Revision Prosthesis (TRP) complications were rare, with chronic fistulized arthritis (2 cases) and recurrent dislocations (2 cases) appearing in the 35–50 and 71–92 age groups.

A chi-square test confirmed a significant association between age group, prosthesis type, and complication type (χ^2^ = 133.834, df = 38, *p* < 0.001), indicating that complication occurrence is influenced by both factors (Table 5). The moderate to strong correlation (Cramer’s V = 0.490) supports the role of age and prosthesis type in complication patterns.

### 3.4. Comorbidities and Their Association with Causes of Complications

The distribution of comorbidities across complication types (idiopathic, infectious, mechanical, and traumatic) is shown in Table 6. Diabetes and metabolic conditions were strongly associated with infections (49 cases, 17.56%), with minimal association with mechanical complications and no cases in idiopathic or traumatic categories.

Obesity and osteoporosis were predominantly linked to traumatic (96 cases, 34.41%) and mechanical complications (57 cases, 20.43%), with moderate associations with infections. Cardiovascular and rheumatic conditions were primarily associated with traumatic complications (96 cases, 34.41%) and also showed a high occurrence of infectious (73 cases, 26.16%) and mechanical complications (56 cases, 20.07%).

Neurological conditions impacted all complication types, most notably traumatic (96 cases, 34.41%), mechanical (56 cases, 20.07%), and idiopathic complications (47 cases, 16.85%), with a smaller proportion associated with infections (30 cases, 10.75%).

A chi-square test confirmed a highly significant association between comorbidities and complication types (*p* < 0.001) (Table 7). Chi-square values ranged from 145.820 to 237.185, with Cramer’s V values between 0.723 and 0.922, indicating strong to very strong associations. Cardiovascular conditions and rheumatism had the strongest associations (Cramer’s V = 0.922), while osteoporosis also showed a high correlation (Cramer’s V = 0.861), particularly with mechanical and traumatic complications.

### 3.5. Association Between Time Intervals and Complications

The distribution of complications across long-term and medium-term intervals is summarized in Table 8.

Infections were more common in the medium-term, with 35 cases in CTP compared to 5 in the long-term. UTP infections were rare (3 cases total). Implant loosening was more frequent in the long-term (20 cases vs. 11 medium-term).

Dislocations were among the most reported complications. In CTP, long-term cases (43) outnumbered medium-term cases (24), while UTP dislocations were more evenly distributed (13 vs. 10 cases). Recurrent dislocations in CTP were more frequent in the long-term (14 vs. 6 medium-term), while UTP cases remained low (2 per interval).

Femur periprosthetic fractures were more frequent in the long-term (20 cases vs. 12 medium-term). CTP periprosthetic fractures were evenly distributed (6 per interval). Hematomas in CTP were more common in the medium-term (7 vs. 3 long-term), while UTP hematomas were slightly more frequent in the long-term (4 vs. 1 medium-term).

Heterotopic ossifications occurred in the long-term for CTP (3 cases), while UTP cases were more evenly distributed (3 vs. 4 cases). Periprosthetic metallosis was more frequent in the long-term (5 vs. 1 medium-term). Prosthetic NACF was rare, with only 1 long-term case.

A chi-square test confirmed a significant association between complication type and time interval (χ^2^ = 58.149, df = 19, *p* < 0.001) (Table 9). Cramer’s V value (0.457) indicates a moderate to strong association.

### 3.6. Treatment Modalities and Their Association with Complications

Table 10 summarizes the distribution of treatment modalities across idiopathic, infectious, mechanical, and traumatic complications. Joint debridement, lavage, and drainage were primarily used for infectious complications, with 45 to 51 cases (16.13–18.28%), and were rarely applied to mechanical and traumatic causes. Reduction and skeletal traction were mainly used for mechanical (35 cases, 12.54%) and traumatic complications (30–33 cases, 10.75–11.83%).

Antibiotics were exclusively used for infections (79 cases, 28.32%), while anti-inflammatory drugs were most associated with idiopathic (6 cases, 2.15%) and infectious complications (5 cases, 1.79%). Osteosynthesis and component extraction were primarily used for traumatic complications (33 cases, 11.83%), with component extraction more frequent in infections (13 cases, 4.66%).

CTP revision was more common in idiopathic (24 cases, 8.60%) and mechanical (15 cases, 5.38%) complications, while UTP overhaul was rarely used (3 cases, 1.08%) for traumatic causes. Physical-kinetic therapy was mostly used for idiopathic complications (6 cases, 2.15%), with limited application in other categories.

A chi-square test confirmed significant associations between treatment modalities and complication causes (Table 11). Joint debridement, lavage, and drainage were significantly associated with infectious complications (χ^2^ = 94.283–94.500, *p* < 0.001, Cramer’s V = 0.581–0.582). Reduction and skeletal traction were moderately associated with mechanical and traumatic complications (χ^2^ = 34.164 and 32.630, *p* < 0.001, Cramer’s V = 0.350 and 0.342).

Antibiotics had the strongest association with infectious complications (χ^2^ = 279.000, *p* < 0.001, Cramer’s V = 1.000). Osteosynthesis was significantly associated with traumatic complications (χ^2^ = 53.731, *p* < 0.001, Cramer’s V = 0.439), while component extraction was linked to infectious and mechanical complications (χ^2^ = 18.243, *p* < 0.001, Cramer’s V = 0.256).

CTP revision was significantly associated with idiopathic and mechanical complications (χ^2^ = 38.579, *p* < 0.001, Cramer’s V = 0.372), while UTP overhaul was not significantly associated with any complication type (χ^2^ = 5.781, *p* = 0.123, Cramer’s V = 0.144). Physical-kinetic therapy showed a weak but significant association across complication types (χ^2^ = 12.238, *p* = 0.007, Cramer’s V = 0.209).

## 4. Discussion

### 4.1. Time Intervals Post-Surgery and the Occurrence and Distribution of Complications

This study confirms a strong association between postoperative time intervals and complication types, with infections predominantly occurring in the medium-term (1–5 years) and mechanical failures, such as dislocations and implant loosening, increasing in the long-term (≥6 years). These findings align with prior research indicating a higher risk of infections in the early postoperative phase and progressive mechanical deterioration over time [28,29,30,31,32].

Comorbidities such as diabetes and obesity have been identified as key risk factors for infection, consistent with these observations [33,34]. This highlights the importance of patient-specific risk assessments and targeted preventive strategies.

Studies have also emphasized the necessity of early detection and monitoring strategies for mechanical failures in the long term. The findings are consistent with this, reinforcing the importance of regular imaging and functional assessments to identify prosthesis-related complications before they lead to severe outcomes [35,36].

While the absence of a matched control group limits direct causative assessments, the retrospective design enables a comprehensive analysis of temporal trends in complication patterns. Future research should consider prospective studies with control groups to better evaluate causative factors and optimize preventive strategies.

Given these findings, implementing time-sensitive postoperative care strategies is crucial. In the medium-term, priority should be given to infection prevention measures, including enhanced wound care, infection control protocols, and early detection strategies. In the long-term, postoperative management should focus on early detection of mechanical complications through regular imaging and functional assessments [36,37,38,39]. These findings reinforce the importance of optimizing infection control protocols, particularly for high-risk patient populations.

While heterotopic ossification was classified within the medium- and long-term intervals, its true temporal onset remains unclear. Future research should adopt more precise time-based analyses to refine complication management strategies.

A structured postoperative monitoring protocol tailored to each recovery phase can improve outcomes by reducing infection-related complications in the medium-term and mechanical failures in the long-term. Implementing timing-specific interventions can enhance patient recovery and reduce complication-related morbidity.

### 4.2. Association Between Treatment Modalities and Specific Types of Complications

This study confirms a strong association between treatment modalities and specific complication types. Antibiotics were exclusively used for infectious complications, while mechanical treatments, such as reduction and skeletal traction, were primarily associated with fractures and dislocations. Procedures like joint debridement, lavage, and drainage were commonly employed for infection management, reinforcing their established role in infection control and joint preservation [40,41,42].

The statistically significant association between antibiotic use and infectious complications highlights the crucial role of timely antibiotic intervention in preventing deep infections and prosthetic failures. This finding is consistent with previous research indicating that early and appropriately targeted antibiotic administration significantly reduces infection-related complications and the need for revision surgery [18,43].

Similarly, for mechanical failures, early surgical intervention has been shown to improve patient outcomes. The effectiveness of mechanical interventions in restoring joint stability aligns with prior evidence supporting the timely reduction and stabilization of fractures and dislocations. Studies have demonstrated that prompt surgical intervention leads to better postoperative mobility, reduced joint instability, and lower rates of revision surgery [44,45,46,47].

Although this study confirms a significant association between antibiotic use and infection control, specific details regarding antibiotic type, dosage, treatment duration, and identified pathogens were not available in the dataset. This limitation restricts a deeper evaluation of optimal antibiotic regimens for different infection profiles. Future studies should focus on identifying the most effective antibiotic regimens by incorporating microbiological data on infection-causing pathogens and resistance patterns.

Despite this limitation, clinical guidelines for Periprosthetic Joint Infections (PJI) recommend empirical broad-spectrum antibiotics, such as cefazolin or vancomycin for Gram-positive bacteria and beta-lactams or fluoroquinolones for Gram-negative infections [48,49]. The absence of pathogen-specific antibiotic data in this study highlights the need for individualized treatment protocols based on microbiological findings to optimize infection management outcomes.

A targeted approach to complication management is critical. Infectious complications should be addressed with timely antibiotic administration, supplemented by joint debridement or lavage, particularly in cases of persistent or deep infections. Mechanical failures require early reduction and stabilization to prevent long-term functional impairment and secondary complications. Individualized treatment plans should consider patient-specific factors, including comorbidities, prior surgeries, and overall health status, to improve clinical outcomes and minimize postoperative risks.

### 4.3. Higher Incidence of Infectious Complications in the Medium-Term

This study confirms that infectious complications occur more frequently in the medium-term (1–5 years) compared to the long-term (≥6 years), aligning with previous findings on early-phase infection risks following joint replacement surgery [50,51]. Infection rates are influenced by multiple factors, including immune response, comorbidities (e.g., diabetes, obesity), surgical techniques, and postoperative care protocols [52,53]. The lower incidence of infections in the long term suggests improvements in postoperative management and infection prevention strategies over time [54,55].

The higher-than-expected rate of septic complications in this study may be due to the inclusion of patients with significant comorbidities and chronic septic complications, as well as the study setting, which involved a referral center for complex cases [56,57]. These findings highlight the importance of targeted infection prevention and management strategies, particularly for high-risk populations.

Vigilant infection monitoring is crucial in the medium-term phase, especially for patients with comorbidities such as diabetes and obesity [58,59,60]. Routine follow-ups, early detection protocols, and patient education on infection symptoms are essential preventive measures. Future research should explore personalized infection prevention strategies to further mitigate medium-term infection risks.

### 4.4. Predominant Use of Antibiotics for Infectious Complications

This study confirms a strong association between antibiotic use and infectious complications, with statistical analysis (Cramer’s V = 1.000) indicating a perfect correlation. These findings reinforce antibiotics as the primary treatment for prosthetic joint infections, aligning with established clinical practices [61,62,63]. Unlike previous studies that primarily assess clinical outcomes, this research provides quantitative evidence supporting the necessity of timely antibiotic administration for infection control [49,64,65].

The results emphasize the importance of standardized antibiotic protocols, ensuring timely, targeted administration based on patient risk factors and comorbidities [66,67,68]. However, the lack of detailed data on antibiotic regimens (e.g., type, duration, and route of administration) limits further analysis regarding the effectiveness of specific antibiotics and the potential impact of prolonged use on antimicrobial resistance. Future research should focus on identifying optimal antibiotic strategies to enhance infection management and improve long-term patient outcomes.

### 4.5. Increase in Mechanical Complications over Time

This study confirms a higher incidence of mechanical complications in the long-term (≥6 years post-surgery), particularly implant loosening and periprosthetic fractures, consistent with previous findings on prosthetic wear and failure over time [69,70]. These results emphasize the need for long-term follow-up and monitoring to preserve joint function and improve patient outcomes [71,72,73].

Periprosthetic fractures primarily occurred around the femoral stem, particularly in the proximal femur, a site vulnerable to stress-related fractures due to high mechanical loading and potential bone resorption. Less commonly, fractures near the acetabular cup were observed, often linked to osteolysis-induced bone loss and implant micromotion. Treatment varied depending on fracture location and implant stability. Stable fractures with well-fixed implants were typically managed with open reduction and internal fixation using plates and cerclage wires, while fractures associated with implant loosening required revision arthroplasty with stem or acetabular component replacement.

A notable limitation is the absence of documented component loosening in uncemented prostheses, despite being a well-recognized long-term issue. This discrepancy may reflect differences in diagnostic or reporting practices or underrepresentation in medical records [74,75,76]. Future prospective studies with standardized reporting protocols are needed to assess uncemented prosthesis outcomes more accurately.

Comorbidities play a significant role in mechanical failures. Diabetes impairs healing and immune function, increasing implant instability and failure risk. Obesity places excess mechanical stress on the prosthesis, accelerating implant wear and loosening [77,78,79,80]. Osteoporosis further raises the likelihood of periprosthetic fractures and aseptic loosening, particularly in postmenopausal women and elderly patients [81,82].

The statistical significance of these associations highlights the importance of patient-specific risk assessments. Diabetes management, weight control, and osteoporosis treatment should be integrated into long-term arthroplasty care to reduce mechanical complications and improve implant longevity.

Prosthesis-related risk factors also contribute to mechanical failures. Uncemented prostheses are more prone to early periprosthetic fractures due to initial implant instability, whereas cemented stems may have a lower early fracture risk but a higher long-term failure rate due to cement degradation. Implant design and surgical technique affect stress distribution, bone integration, and stability, warranting careful selection in high-risk patients [83,84,85].

Advancements in prosthetic materials and surgical techniques could help mitigate long-term mechanical risks. Modern prostheses with improved durability and fixation methods offer potential benefits over traditional designs, warranting further investigation [86,87,88].

Long-term follow-up should include regular imaging, such as X-rays and DEXA scans, to detect early mechanical failures. Individualized care plans should address patient comorbidities, with targeted interventions such as weight management for obesity and bisphosphonate therapy for osteoporosis to improve bone health and reduce fracture risk.

### 4.6. Future Research Directions

Future research should prioritize longitudinal and prospective studies to track complication progression, treatment effectiveness, and late-emerging issues such as prosthesis wear and infection recurrence. Specific conditions like heterotopic ossification require timing-based evaluations to determine the onset, risk factors, and clinical outcomes. Multicenter studies with diverse populations are essential for improving generalizability and accounting for variations in complication rates and treatment efficacy across healthcare systems.

Further investigations should explore demographic, lifestyle, and genetic risk factors to develop personalized prevention strategies. Emerging technologies, including robotic-assisted surgery, 3D printing, and advanced prosthetic materials, warrant evaluation to determine their impact on prosthesis durability, mechanical failure rates, and infection risks. Additionally, artificial intelligence and predictive modeling could improve risk assessment, preoperative planning, and postoperative monitoring.

The psychosocial impact of complications remains underexplored, particularly regarding mental health, anxiety, depression, and quality of life. Understanding these effects can support integrated care models that incorporate psychological support alongside surgical management. Future studies should also assess the comparative effectiveness of treatment modalities, refining best practices for antibiotics, joint debridement, and mechanical stabilization.

Health economic evaluations are needed to assess the cost-effectiveness of early mechanical interventions and infection prevention strategies, guiding healthcare policy and resource allocation. Research should also explore patient education strategies, digital tools, and telemedicine to improve patient engagement and long-term monitoring.

Future research should explicitly investigate complications occurring within the immediate postoperative period (<1 year), particularly PJI, periprosthetic fractures, and dislocations, given their prominence in registry data and changing patterns over recent decades. Analyzing these early complications in conjunction with medium- and long-term outcomes could provide a more comprehensive understanding of postoperative risks, informing tailored preventive strategies and early management protocols.

Furthermore, future studies should aim to include larger, multi-center cohorts to validate the findings of this study and ensure the robustness of the conclusions across diverse populations and healthcare settings. Larger sample sizes will allow for more reliable statistical analyses and a deeper understanding of the patterns of complications in hip arthroplasty patients.

Additionally, future studies should consider stratifying patient cohorts by different time periods to account for the significant changes in prosthesis types, perioperative protocols, and surgical techniques over time. This approach would allow for a clearer understanding of how advancements in these areas have influenced complication rates and long-term outcomes. Stratification can help reduce the potential confounding effects of historical changes in surgical practice and prosthetic design.

Addressing these gaps will refine clinical decision-making, optimize personalized treatment strategies, and enhance postoperative care, ultimately reducing complications and improving long-term patient outcomes.

### 4.7. Implications for Practice

This study highlights the importance of time-sensitive postoperative monitoring and treatment strategies in managing hip arthroplasty complications. Medium-term care (1–5 years) should focus on infection prevention and early detection, using routine clinical follow-ups, laboratory tests, and patient education on infection symptoms. In the long-term (≥6 years), monitoring should shift toward detecting mechanical complications, such as dislocations, implant loosening, and fractures, through regular imaging techniques (X-rays, CT scans).

Treatment strategies should align with the complication type. Infectious complications require timely antibiotic administration, often supplemented by joint debridement or lavage, while mechanical failures should be managed with early surgical interventions, including reduction and stabilization to restore function and prevent further deterioration. Individualized treatment plans should consider patient-specific factors, such as comorbidities and surgical history, to optimize outcomes.

Infection prevention should extend beyond the immediate postoperative period, emphasizing proper wound care, prophylactic antibiotics, and patient education, particularly during the medium-term phase, when infection risk is highest. Long-term follow-up should integrate regular imaging and functional assessments to detect early signs of mechanical failures and enhance prosthesis longevity.

Comorbidities such as diabetes, obesity, and osteoporosis significantly influence complication risks. Targeted interventions, including weight management for obese patients and bisphosphonate therapy for osteoporosis, can reduce joint strain and fracture risk.

A multidisciplinary approach involving orthopedic surgeons, primary care providers, physical therapists, and infection control specialists is essential for comprehensive long-term care. Patient engagement through education and communication improves adherence to postoperative protocols, lifestyle modifications, and follow-up visits, ultimately reducing complication rates.

Integrating these strategies into clinical practice can improve patient outcomes, reduce complications, and enhance long-term quality of life. Future studies should validate these recommendations in diverse populations and healthcare settings to refine best practices and improve applicability.

### 4.8. Limitations of the Study

While this study provides insights into the temporal patterns of complications and their treatment associations following hip arthroplasty, several limitations must be acknowledged.

The retrospective design limits the ability to establish causal relationships between treatment modalities and complication outcomes. Additionally, retrospective studies are inherently susceptible to recall bias and missing data, potentially affecting the accuracy of recorded complications. Future prospective cohort studies with standardized data collection can provide stronger causal inferences and minimize bias.

This single-center study limits generalizability, as variations in surgical techniques, implant types, and postoperative care protocols may influence complication rates. Additionally, although our cohort consisted of 279 patients, which is a relatively small sample size, a larger, multi-center cohort would provide more robust conclusions and improve the external validity of our findings. Future studies with larger, more diverse patient populations are essential to validate our results and enhance the applicability of the findings across different healthcare settings.

Several potential confounding variables were not accounted for, including smoking status, nutritional deficiencies, and surgeon experience, which may have influenced complication rates. Future research should integrate broader patient- and surgery-related factors to refine these associations.

The lack of detailed antibiotic data (e.g., specific agents, treatment duration, and administration routes) limits a nuanced assessment of infection management effectiveness. Without this information, it is difficult to determine which antibiotic regimens were most successful in preventing deep infections. Future studies should collect comprehensive antibiotic regimen data to optimize infection control strategies.

Additionally, this study focused primarily on medium- and long-term complication intervals without extended lifetime follow-up, limiting insights into how complications evolve over time. Prospective longitudinal studies can help determine the long-term effectiveness of treatment modalities and complication progression.

A notable limitation is the absence of documented component loosening in uncemented prostheses, despite being a well-recognized long-term complication. This likely reflects reporting inconsistencies or diagnostic variability rather than the actual absence of implant loosening. Implementing standardized reporting protocols would improve the accuracy of long-term implant stability assessments.

Variability in treatment protocols across institutions, such as differences in antibiotic use, surgical techniques, and mechanical interventions, was not considered. Future studies should compare standardized versus individualized treatment protocols to enhance consistency and optimize best practices.

Moreover, this study did not include complications occurring within the first postoperative year, such as early PJI, periprosthetic fractures, and dislocations, which are among the most commonly reported complications in joint registries and literature. By starting our analysis at one year postoperatively, we potentially omitted critical insights into the early postoperative complication patterns, particularly since the relative frequency and importance of these complications have been changing over time according to registry reports. Future studies should incorporate the immediate postoperative period (<1 year) to fully capture the early onset and progression of complications, enabling a comprehensive evaluation across all recovery phases.

Lastly, this study focused on infections, mechanical failures, and dislocations but did not assess chronic pain, psychosocial impacts, or rare complications, which can also significantly impact long-term outcomes. A broader research scope is necessary to capture the full range of post-surgical risks and their impact on quality of life.

Acknowledging these limitations helps contextualize the findings and guide future research directions. Addressing these gaps will strengthen clinical evidence, refine surgical protocols, and improve patient outcomes in hip arthroplasty.

## 5. Conclusions

This study provides critical insights into the temporal distribution of complications following hip arthroplasty and their association with treatment modalities. Findings confirm that infections are more prevalent in the medium-term (1–5 years), while mechanical complications, including dislocations, implant loosening, and fractures, increase in the long-term (≥6 years). A strong association between treatment choice and complication type was observed, with antibiotics predominantly used for infections and mechanical interventions, such as joint debridement and reduction, addressing structural failures.

These findings emphasize the importance of time-sensitive postoperative management. The higher incidence of infections in the medium-term highlights the need for enhanced monitoring and infection prevention protocols, particularly in high-risk populations. The increasing prevalence of mechanical failures over time underscores the necessity of long-term follow-up and early detection strategies to prevent prosthetic failure.

By reinforcing the importance of tailored treatment strategies, this study strengthens the evidence base for optimizing hip arthroplasty care. Future research should focus on addressing existing limitations, exploring additional risk factors, and developing strategies to mitigate long-term complications. Advancing this field is essential for improving surgical outcomes, refining treatment protocols, and enhancing long-term patient care.

## Figures and Tables

**Table 1:** Distribution of Participants by Gender and Age Group.

Variable	Level	Frequency	Percent
Gender	Female	157	56.27%
	Male	122	43.73%
Age	35–50 years	12	4.30%
	51–70 years	124	44.44%
	71–92 years	143	51.26%
Total		279	100.00%

**Table 2:** Distribution of Complications by Gender in Total Hip Arthroplasty.

	Gender		
Complication	Female	Male	Total
CTP chronic fistulized arthritis	3	2	5
CTP implant loosening	18	13	31
CTP dislocation	46	21	67
CTP heterotopic ossifications	2	1	3
CTP infection	18	22	40
CTP periprosthetic fracture	8	4	12
CTP post-traumatic hematoma	9	1	10
CTP recurrent dislocation	15	5	20
Femur periprosthetic fracture	11	21	32
Periprosthetic metallosis	4	2	6
Prosthetic NACF	1	0	1
TRP chronic fistulized arthritis	0	2	2
TRP recurrent dislocation	2	0	2
UTP chronic fistulized arthritis	0	4	4
UTP dislocation	11	12	23
UTP heterotopic ossifications	1	6	7
UTP infection	1	2	3
UTP periprosthetic fracture	3	0	3
UTP post-traumatic hematoma	2	3	5
UTP recurrent dislocation	2	1	3
Total	157	122	279

CTP—Cemented Total Prosthesis; NACF—Non-Articular Collapsing Fracture; TRP—Total Revision Prosthesis; UTP—Uncemented Total Prosthesis.

**Table 3:** Chi-Square Test of Independence for Gender and Complications Following Total Hip Arthroplasty.

	Value	df	*p*	Cramer’s V
Χ^2^	40.319	19	0.003	0.380
*N*	279			

**Table 4:** Distribution of Complications by Age Group and Prosthesis Type.

	Age			
Complication	35–50	51–70	71–92	Total
CTP chronic fistulized arthritis	0	2	3	5
CTP implant loosening	0	9	22	31
CTP dislocation	1	23	43	67
CTP heterotopic ossifications	0	0	3	3
CTP infection	1	16	23	40
CTP periprosthetic fracture	0	7	5	12
CTP post-traumatic hematoma	1	6	3	10
CTP recurrent dislocation	0	11	9	20
Femur periprosthetic fracture	1	22	9	32
Periprosthetic metallosis	0	1	5	6
Prosthetic NACF	0	1	0	1
TRP chronic fistulized arthritis	0	0	2	2
TRP recurrent dislocation	2	0	0	2
UTP chronic fistulized arthritis	0	2	2	4
UTP dislocation	3	15	5	23
UTP heterotopic ossifications	0	0	7	7
UTP infection	0	1	2	3
UTP periprosthetic fracture	2	1	0	3
UTP post-traumatic hematoma	1	4	0	5
UTP recurrent dislocation	0	3	0	3
Total	12	124	143	279

CTP—Cemented Total Prosthesis; NACF—Non-Articular Collapsing Fracture; TRP—Total Revision Prosthesis; UTP—Uncemented Total Prosthesis.

**Table 5:** Chi-Square Test Results for Association Between Age Group, Prosthesis Type, and Complication Type.

	Value	df	*p*	Cramer’s V
Χ^2^	133.834	38	<0.001	0.490
*N*	279			

**Table 6:** Association Between Comorbidities and Causes of Complications.

Variable	Complication Cause	Frequency	Percentage
Diabetes	Idiopathic	0	0.00%
	Infectious	49	17.56%
	Mechanical	1	0.36%
	Traumatic	0	0.00%
Obesity	Idiopathic	47	16.85%
	Infectious	30	10.75%
	Mechanical	57	20.43%
	Traumatic	96	34.41%
Osteoporosis	Idiopathic	0	0.00%
	Infectious	24	8.60%
	Mechanical	57	20.43%
	Traumatic	96	34.41%
Cardiovascular	Idiopathic	0	0.00%
	Infectious	73	26.16%
	Mechanical	56	20.07%
	Traumatic	96	34.41%
Neurological	Idiopathic	47	16.85%
	Infectious	30	10.75%
	Mechanical	56	20.07%
	Traumatic	96	34.41%
Metabolic	Idiopathic	0	0.00%
	Infectious	49	17.56%
	Mechanical	1	0.36%
	Traumatic	0	0.00%
Rheumatism	Idiopathic	0	0.00%
	Infectious	73	26.16%
	Mechanical	56	20.07%
	Traumatic	96	34.41%

**Table 7:** Chi-Square Test Results for Association Between Comorbidities and Causes of Complications.

Variable	Χ^2^ Value	df	*p*	Cramer’s V
Diabetes	145.820	3	<0.001	0.723
Obesity	150.479	3	<0.001	0.734
Osteoporosis	206.959	3	<0.001	0.861
Cardiovascular	237.185	3	<0.001	0.922
Neurological	145.820	3	<0.001	0.723
Metabolic	145.820	3	<0.001	0.723
Rheumatism	237.185	3	<0.001	0.922

**Table 8:** Chi-Square Test for the Association Between Complications and Time Intervals.

	Time Interval		
Complication	Long-Term Interval (≥6 Years)	Medium-Term Interval (1–5 Years)	Total
CTP chronic fistulized arthritis	0	5	5
CTP implant loosening	20	11	31
CTP dislocation	43	24	67
CTP heterotopic ossifications	3	0	3
CTP infection	5	35	40
CTP periprosthetic fracture	6	6	12
CTP post-traumatic hematoma	3	7	10
CTP recurrent dislocation	14	6	20
Femur periprosthetic fracture	20	12	32
Periprosthetic metallosis	5	1	6
Prosthetic NACF	1	0	1
TRP chronic fistulized arthritis	0	2	2
TRP recurrent dislocation	2	0	2
UTP chronic fistulized arthritis	2	2	4
UTP dislocation	13	10	23
UTP heterotopic ossifications	3	4	7
UTP infection	0	3	3
UTP periprosthetic fracture	2	1	3
UTP post-traumatic hematoma	4	1	5
UTP recurrent dislocation	2	1	3
Total	148	131	279

CTP—Cemented Total Prosthesis; NACF—Non-Articular Collapsing Fracture; TRP—Total Revision Prosthesis; UTP—Uncemented Total Prosthesis.

**Table 9:** Chi-Square Test Results for Complications Across Time Intervals.

	Value	df	*p*	Cramer’s V
Χ^2^	58.149	19	<0.001	0.457
*N*	279			

**Table 10:** Frequency and Percentage of Complication Causes for Various Treatment Modalities.

Variable	Complication Cause	Frequency	Percentage
Joint debridement	Idiopathic	4	1.43%
	Infectious	45	16.13%
	Mechanical	1	0.36%
	Traumatic	6	2.15%
Lavage	Idiopathic	5	1.79%
	Infectious	51	18.28%
	Mechanical	3	1.08%
	Traumatic	10	3.58%
Drainage	Idiopathic	5	1.79%
	Infectious	51	18.28%
	Mechanical	3	1.08%
	Traumatic	10	3.58%
Hematoma evacuation	Idiopathic	0	0.00%
	Infectious	2	0.72%
	Mechanical	2	0.72%
	Traumatic	4	1.43%
Reduction	Idiopathic	10	3.58%
	Infectious	13	4.66%
	Mechanical	35	12.54%
	Traumatic	30	10.75%
Skeletal traction	Idiopathic	11	3.94%
	Infectious	13	4.66%
	Mechanical	35	12.54%
	Traumatic	33	11.83%
Antibiotics	Idiopathic	0	0.00%
	Infectious	79	28.32%
	Mechanical	0	0.00%
	Traumatic	0	0.00%
Anti-inflammatory	Idiopathic	6	2.15%
	Infectious	5	1.79%
	Mechanical	2	0.72%
	Traumatic	1	0.36%
Osteosynthesis	Idiopathic	2	0.72%
	Infectious	2	0.72%
	Mechanical	1	0.36%
	Traumatic	33	11.83%
Component extraction	Idiopathic	1	0.36%
	Infectious	13	4.66%
	Mechanical	4	1.43%
	Traumatic	1	0.36%
CTP Revision	Idiopathic	24	8.60%
	Infectious	4	1.43%
	Mechanical	15	5.38%
	Traumatic	17	6.09%
UTP overhaul	Idiopathic	0	0.00%
	Infectious	0	0.00%
	Mechanical	0	0.00%
	Traumatic	3	1.08%
Physical-kinetic therapy	Idiopathic	6	2.15%
	Infectious	2	0.72%
	Mechanical	2	0.72%
	Traumatic	1	0.36%

CTP—Cemented Total Prosthesis; UTP—Uncemented Total Prosthesis.

**Table 11:** Chi-Square Test Results for the Association Between Treatment Modalities and Causes of Complications.

Variable	Χ^2^ Value	df	*p*	Cramer’s V
Joint debridement	94.283	3	<0.001	0.581
Lavage	94.500	3	<0.001	0.582
Drainage	94.500	3	<0.001	0.582
Hematoma evacuation	2.085	3	0.555	0.086
Reduction	34.164	3	<0.001	0.350
Skeletal traction	32.630	3	<0.001	0.342
Antibiotics	279.000	3	<0.001	1.000
Anti-inflammatory	9.662	3	0.022	0.186
Osteosynthesis	53.731	3	<0.001	0.439
Component extraction	18.243	3	<0.001	0.256
CTP Revision	38.579	3	<0.001	0.372
UTP overhaul	5.781	3	0.123	0.144
Physical-kinetic therapy	12.238	3	0.007	0.209

CTP—Cemented Total Prosthesis; UTP—Uncemented Total Prosthesis.

## Data Availability

The raw data that supports the findings of this article can be provided by the authors upon request.

## Abbreviations

The following abbreviations are used in this manuscript:

NACF	Non-Articular Collapsing Fractures
CTP	Cemented Total Prosthesis
TRP	Total Revision Prosthesis
UTP	Uncemented Total Prosthesis
PJI	Periprosthetic Joint Infections
